# Mixed Medullary-Follicular Carcinoma of the Thyroid

**DOI:** 10.1155/2013/571692

**Published:** 2013-10-29

**Authors:** Maasumeh Tohidi, Gholamreza Pourbehi, Mohammad Bahmanyar, Seyed Sajjad Eghbali, Mohammadreza Kalantar Hormozi, Iraj Nabipour

**Affiliations:** ^1^The Persian Gulf Marine Medicine Biotechnology Research Center, Department of Endocrinology, Bushehr University of Medical Sciences, Bushehr, Iran; ^2^The Persian Gulf Marine Medicine Biotechnology Research Center, Bushehr University of Medical Sciences, Bushehr, Iran; ^3^The Persian Gulf Marine Medicine Biotechnology Research Center, Department of Pathology, Bushehr University of Medical Sciences, Bushehr, Iran

## Abstract

*Introduction*. Mixed medullary-follicular thyroid carcinoma is an uncommon tumor that consists of both follicular and parafollicular cells. *Case*. We report a 43-year-old woman with a palpable mass in the right side of the neck. Fine needle aspiration suggested a diagnosis of high grade anaplastic carcinoma that has been associated with papillary features. Total thyroidectomy was done in which histopathological examination showed diagnosis of medullary carcinoma. Immunohistochemical staining was positive for chromogranin, calcitonin, and thyroglobulin in tumoral cells. *Conclusion*. Mixed medullary-follicular thyroid carcinoma is a rare tumor. Diagnosis of these tumors with fine needle aspiration is very difficult and may lead to misdiagnosis. It is necessary to correlate the cytological finding with serum calcitonin and thyroglobulin. Also immunostaining for calcitonin and thyroglobulin confirms diagnosis.

## 1. Introduction

Medullary thyroid carcinoma (MTC) is a neuroendocrine tumor that originates from parafollicular or c-cells of the thyroid gland [1]. MTC accounts for 5–10% of all thyroid carcinomas. A characteristic feature of this tumor is production of calcitonin. Most MTCs (80%) are sporadic and others (20%) are familial [2, 3]. Pfaltz et al. in 1959 reported that MTC is a tumor with solid-nonfollicular pattern, and it is different from other thyroid carcinomas clinically and pathologically [4]. Also it was noted that histological appearance of MTC may be atypical, and follicular structures can be seen in it. Also it was shown that these tumors have positive immunoreactivity for calcitonin and thyroglobulin. Subsequently such tumors were named mixed medullary-follicular carcinoma [4, 5].

Mixed medullary-follicular carcinoma is a rare tumor of the thyroid. Less than 40 cases have been reported in the literature [4–8].

The cellular origin of the mixed medullary-follicular tumors is unknown.

Here we report a particular presentation of a mixed medullary-follicular carcinoma in which fine needle aspiration showed anaplastic carcinoma and mixed medullary-follicular thyroid carcinoma confirmed after thyroidectomy.

## 2. Case

A 43-year-old woman presented with a palpable mass in the right side of the neck, increasing in size over several months. Physical examination revealed a 4 cm  ×  4 cm, well-defined nontender nodule in right thyroidal lobe. There were no other physical abnormalities. The patient didnot smoke. Her family history was negative for thyroid and other endocrine tumors. The patient had no history of neck or whole body radiation. The plasma levels of T_3_, T_4_, and TSH were within normal limits. Fine needle aspiration showed cells with large nucleolus and severe pleomorphism with combined follicular and papillary like pattern. Pathologist suggested diagnosis of the high grade anaplastic carcinoma with papillary pattern. The total thyroidectomy was performed.

*Macroscopic Finding*. The surgical specimen was fixed in buffered formalin. The cut surface of thyroid gland showed a well-defined nodule measuring 4 cm × 3 cm that was unencapsulated. No area of necrosis and hemorrhage was observed. An other part of thyroid gland was free of lesion.

### 2.1. Microscopic Finding

**Figure 1:**
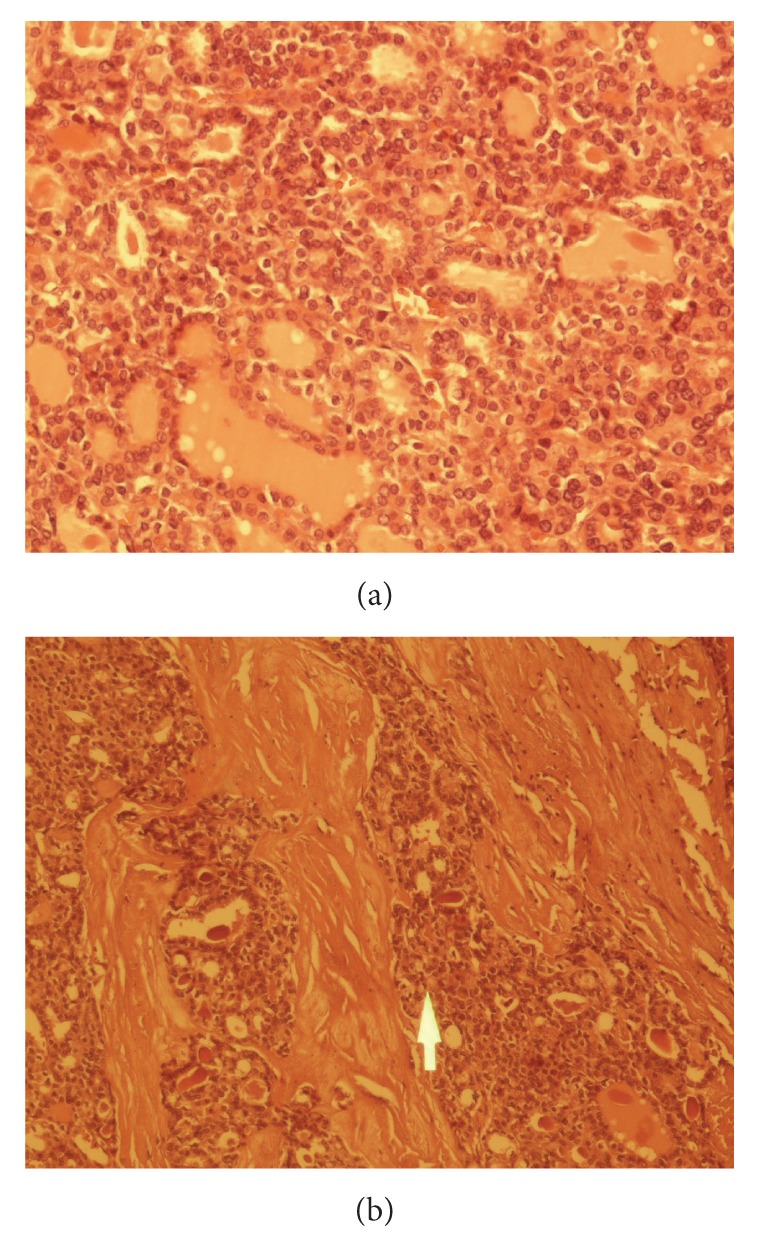
(a) A follicular structures in follicular carcinoma area composed of tumoral cells. (b) Oval polygonal shaped tumoral cells in medullary carcinoma area (arrow).

Several cut sections were stained with hematoxylin and eosin. Solid nests of pleomorphic cells with mitotic activity were seen. Histochemically, there was no evidence of amyloid deposition by using crystal violet (Figure 1). Immunoperoxidase staining was positive for calcitonin, chromogranin, and thyroglobulin.

## 3. Discussion

MTC accounts for 5% to 10% of all thyroid malignancies. It originates from the parafollicular or c-cells of the thyroid gland. Tumoral cells typically produce calcitonin. On histological examination, MTC is composed of cells that vary in morphologic feature and rearrangement. Round, polyhedral, spindle shape cells were described. An amyloid stroma is commonly present. Lymphatic and vascular invasion may be seen. Definitive diagnosis can be confirmed by positive immunostaining of tumor tissue for calcitonin and carcinoembryonic antigen (CEA) [9, 10].

Mixed medullary-follicular carcinoma is composed of neoplastic cells with combined histological and immunohistochemical features of both follicular and parafollicular cells. [5–7, 11–14]. The cellular origin of the mixed medullary-follicular carcinoma is unknown, and several hypotheses were described. One of the hypotheses explains that this tumor might arise from the multipotent stem cells that can be differentiated to follicular and c-cells [7, 15, 16].

An other hypothesis shows that common oncogenic stimulus may affect both follicular and parafollicular cells [7, 17].

Ectopic production of thyroglobulin in classical MTCs has been described. Also, additional molecular alternation in MTC may be involved in differentiation of MTC cells toward a follicular phenotype. Volante et al. reported hostage hypothesis. Neoplastic transformation of c-cells leads to the development of MTC with entrapment of normal follicles among cancerous cells. The microenvironment provided by MTC cells would subsequently stimulate the proliferation of the trapped follicular cells into the tumoral phenotype [18].

In some studies, RET protooncogene mutation might be involved [11, 19].

Review of literature showed less than 50 cases of mixed medullary-follicular carcinoma. These tumors usually occur in middle age people, and initial presentation of them is swelling of the neck. Serum calcitonin and thyroglobulin are helpful markers in diagnosis, because calcitonin is produced from medullary component and thyroglobulin from follicular cells. The size of this tumor usually is 1 cm to 5/5 cm. There are unifocal tumors while multifocal tumors are particularly associated with MEN type IIA. In most patients lymph node metastasis is present at the time of diagnosis [6]. Distant metastasis can be observed frequently in lungs, liver, mediastinum, and bone. These patients might die within 10 years after the diagnosis [6, 11].

In our case, fine needle aspiration was not capable of diagnosing mixed medullary-follicular carcinoma. It seems that role of fine needle aspiration in the diagnosis of this tumor is very limited. In suspicious cases, measurement of the serum calcitonin and thyroglobulin levels is helpful. We should correlate cytological finding with serum calcitonin and thyroglobulin. Also immunostaining for calcitonin and thyroglobulin is essential for confirmation of diagnosis. In our case immunostaining for calcitonin and thyroglobulin was positive.

The principal treatment of mixed medullary-follicular carcinoma is surgery, and total thyroidectomy should be done. Radioiodine ablation is not effective in MTC, but, due to presence of follicular component in mixed medullary-follicular carcinoma, several studies suggested it. A precise diagnosis of this tumor is fundamental for treatment and follow-up [8, 20, 21].
